# Effect of Rock Crystal Addition on the Properties of Silicone Pressure-Sensitive Adhesives

**DOI:** 10.3390/polym17192687

**Published:** 2025-10-04

**Authors:** Adrian Krzysztof Antosik, Marcin Bartkowiak

**Affiliations:** Department of Organic Chemical Technology and Polymer Materials, Faculty of Chemical Technology and Engineering, West Pomeranian University of Technology in Szczecin, Piastów Ave. 42, 71-065 Szczecin, Poland

**Keywords:** rock crystal, filler, silicone pressure-sensitive adhesive, self-adhesive properties, adhesion

## Abstract

In the presented work, a natural mineral—rock crystal—was used as a filler to obtain new silicone adhesive tapes. It was expected that, properly crushed, this hard mineral, consisting almost entirely of silica (silicon dioxide), should enhance the thermal resistance and cohesion of the self-adhesive composition with no/or low reduction in the rest of performance properties of the products. For this purpose, tests were conducted on the functional properties of new self-adhesive tapes, such as adhesion, cohesion, and tack. The obtained results confirmed the scientific assumptions and the thermal resistance of adhesive layers reached over 225 °C. The material itself turned out to not agglomerate in the adhesive composition and to be compatible with it. The new self-adhesive materials have application potential and can be used as materials for special applications in the field of heating, e.g., in connecting pipes, where thermal resistance and thermal expansion are of immense importance.

## 1. Introduction

Adhesives have become essential in diverse industries, offering distinctive properties that cater to various applications. Adhesives can be divided into groups according to various factors. Depending on the type (group) of adhesives, they are characterized by the origin (nature of raw materials used during production) and different properties (adhesion, cohesion, shrinkage), bonding methods (chemical, physical), application and curing methods (thermal curing, UV-curing or applying ready-made compositions). One of the commonly used distinctions between adhesives is the division into liquid or semi-liquid adhesives and pressure-sensitive adhesives (PSAs or PSAs) [1].

Pressure-sensitive adhesives (PSAs) have become integral to numerous industries, offering easier-to-apply solutions to the systems used so far, e.g., connecting with rivets. PSAs are so-called everlasting adhesives, which means that they retain their adhesive properties throughout their use. PSA can be easily applied to surfaces where they do not create permanent (chemical) bonds, and can be relatively easily removed, which distinguishes them from conventional adhesives [2,3]. Among them, silicone pressure-sensitive adhesives (Si-PSAs) have deserved special attention in the last twenty years.

Silicone pressure-sensitive adhesives stand as materials for special applications in the realm of adhesive technology, wielding a plethora of distinctive characteristics that set them apart in applications [4]. Their versatile nature and exceptional performance have rendered them indispensable across a spectrum of industries. At the molecular level, silicone PSAs boast a unique chemistry that imparts them with high tackiness and adherence to various substrates (materials with different—often very low—surface energy). This inherent property makes them particularly adept at forming robust bonds under the influence of minimal pressure, a quality that underpins their classification as pressure-sensitive adhesives. The silicone backbone contributes to their adhesive prowess but also endows them with desirable traits such as resistance to extreme temperatures, UV radiation, and harsh environmental conditions [4,5]. In the electronics sector, these adhesives find utility in bonding delicate components, ensuring secure connections while also providing thermal management. The medical field benefits from their biocompatibility, enabling their use in medical devices and transdermal applications [6,7]. Beyond their functional attributes, silicone PSAs play a pivotal role in enhancing manufacturing efficiency. Their ease of application, coupled with the ability to conform to irregular surfaces, simplifies assembly processes and promotes precision in bonding. This makes them ideal candidates for applications where intricate designs and complex geometries are prevalent. Furthermore, the adhesive landscape continually evolves with research and development efforts aimed at fine-tuning the properties of silicone Si-PSAs [8]. In conclusion, silicone pressure-sensitive adhesives emerge as stalwart contributors to modern industrial processes, underpinning advancements in technology and manufacturing. Their unique amalgamation of properties positions them at the forefront of adhesive innovation, ensuring that they remain indispensable in an ever-expanding array of applications across diverse sectors [9].

Rock crystals, also known as clear quartz or mountain crystal, are a variety of mineral quartz (silicon dioxide). It is characterized by impeccable cleanliness and strong shine. It is one of the most common varieties of quartz produced, among others, in hydrothermal conditions. It is an important rock-forming mineral in igneous, metamorphic, and sedimentary pegmatites. It is a mineral that is extremely resistant to acids and alkalis. It is only dissolved in hydrofluoric acid. It exhibits piezoelectric properties (the ability to generate an electric charge under pressure)—which is used in practical applications in various industries, from electronics to timekeeping devices [10,11,12].

The main goal of the studies was to increase the long-term thermal resistance of the selected silicone pressure-sensitive adhesive. Si-PSA has high thermal resistance on its own, compared to other types of PSAs, but it can be increased using additional mineral fillers. It was discovered during our previous studies that some mineral fillers can significantly increase thermal stability of pressure-sensitive adhesive layers [13,14,15,16]. Introduction of such fillers gives additional benefits such as decrease in smoke generation and increase in fire retardancy in case of fire [17,18,19]. The work presents the modification of a selected self-adhesive silicone resin with the addition of rock crystal. Its aim was to investigate the effect of the addition of filler on the functional and thermal properties of the obtained self-adhesive tapes. The introduction of rock crystal to the self-adhesive silicone resin was also worth examining the influence of its addition on the thermal resistance of the obtained silicone self-adhesive adhesives. Moreover, the shrinkage of the prepared Si-PSA was determined.

## 2. Materials and Methods

### 2.1. Materials

**Figure 1:**
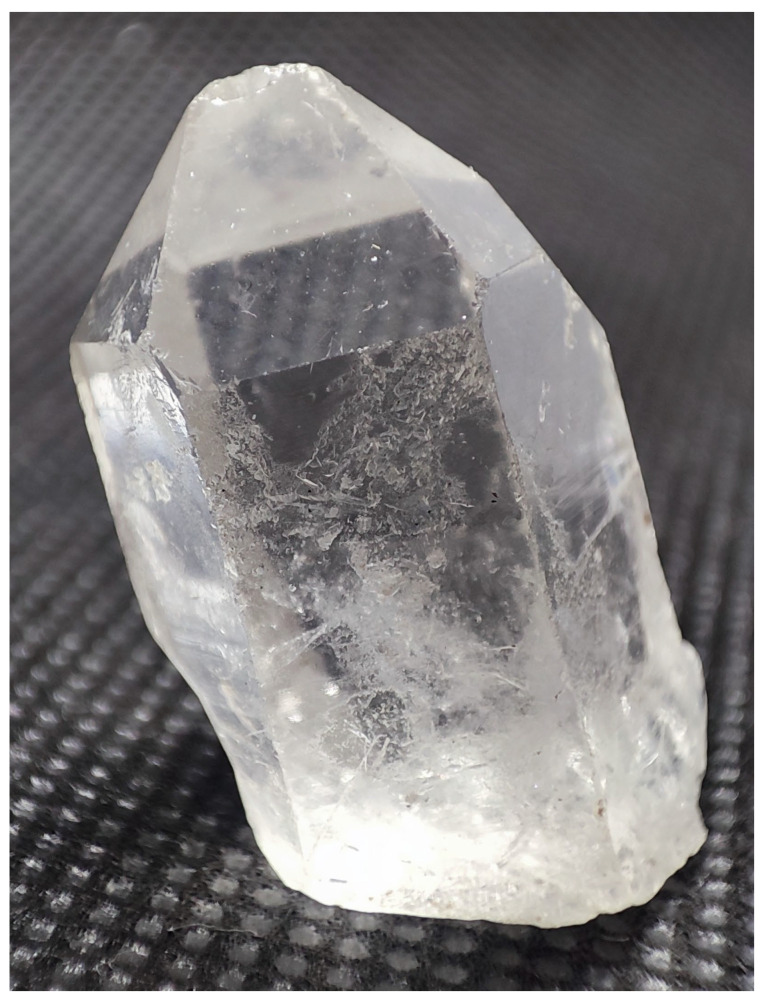
Rock crystal raw material before grinding.

The following materials were used to create this paper:

- Silicone adhesive from Dow Silicones Corp. (Midland, MI, USA)—resin DOWSIL^TM^ 7355 (Q2-7355);
- Crosslinking agent—NOVIPER DB 50—Bis(2,4-dichlorobenzoyl) peroxide (DClBPO) from Novichem Ltd. (Chorzów, Poland);
- Rock crystal (raw material; Figure 1) from Poppystones (Sztutowo, Poland).

Rock crystal used as a filler was ground into dust using a laboratory grinder, with speed of 28,000 rpm, using 50 g portions of mineral and grinding them for 10 min each. The particle size distribution of obtained filler was evaluated using a laser diffraction particle size analyzer Malvern Mastersizer 2000 (Malvern Panalytical Ltd., Malvern, UK). The following characteristic values for particle size distribution were determined and are presented in Table 1: the equivalent diameters of filler particles (D_0.5_, D_0.9_), surface weighted mean D_3.2_, and volume weighted mean D_4.3_.

### 2.2. Preparation of One-Side Self-Adhesive Tapes

Q2-7355 silicone resin crosslinked with 1.5 pph of 2–4-dichlorobenzoyl peroxide (DClBPO) was selected for testing due to its highest performance properties (Table 2). In order to obtain single-sided adhesive tapes, the selected composition was modified with the appropriate addition of filler of 0.1, 0.5, 1.0, and 3.0 pph, respectively (in relation to the mass of the polymer). Each of the homogenized compositions was coated onto a polyester film with a thickness of 50 µm using a semi-automatic laboratory coater. The adhesive film (basis weight of 45 g/m^2^) was then placed in a drying chamber for 10 min at 110 °C to crosslink the adhesive film. Thus, the obtained adhesive film was covered with a second layer of fluorosilicone-coated polyester film (Dolpap Ltd., Chojnów, Poland) and cut into strips for the application tests.

### 2.3. Methods

#### 2.3.1. Pot Life

Pot life, known also as shelf life, is stability during storage of prepared PSA compositions. It is defined as the maximum period of PSA resin storage during which the resin or composition retains its rheological properties and is fully usable for coating. It is also determined by the time it takes to significantly increase the viscosity of the mixture (twice to four times). The tests were proceeded at room temperature and started immediately after preparation of filler–resin composition. Viscosity measurements were taken using the spindle method and IKA Rotavisc me-vi Digital Viscometer (IKA-Werke GmbH & Co. KG, Staufen im Breisgau, Germany).

#### 2.3.2. Adhesion

Adhesion is defined as the interaction between two surfaces of dissimilar substances or phases. This is closely related to the action of intermolecular interactions at the interface of both materials and is one of the most important phenomena occurring when bonding materials. The work test was performed due to the standard developed by FINAT (Fédération Internationale des Fabricants et Transformateurs d’adhesifs et thermocollants sur papiers et autres support) [20]. Standard FTM 1—“Peel adhesion (180°) at 300 mm per minute” has the following test procedure. Samples of PSA tapes are cut in the form of strips 25 × 175 mm and partially attached to the standardized test surface (i.e., steel plate). A tensile testing machine is used to measure the force required to peel off the tape from the steel at a specified angle and with a defined speed. Zwick/Roell Z-25 (Zwick/Roell GmbH & Co. KG, Ulm, Germany) testing machine was used for the presented studies.

#### 2.3.3. Cohesion

Cohesion, also defined as shear strength, is the main factor influencing the durability of the adhesive bond, and is the second most important feature of self-adhesive tapes and adhesives. Cohesion is related to the internal consistency of the adhesive structure, and its value is influenced by, among others, temperature, the concentration and type of crosslinking agent used, and the thickness of the adhesive film. Shear strength is measured according to industrial standard FINAT FTM 8—“Resistance of shear from a standard surface” with the following test procedure [20]. A strip of PSA-coated material is attached to the steel plate with a contact area of 25 × 25 mm. The plate with the attached tape is suspended from a rack placed in the thermostatic chamber. The free end of the PSA tape strip is loaded with a hanging weight of 1 kg. The time is measured until the tape is separated from the plate by a broken joint. The measurements were carried out at 23 °C and 70 °C, using a standardized testing chamber with appropriate racks, hooks, and sensors.

#### 2.3.4. Tack

Tack indicates the ability of an adhesive to adhere briefly without pressure during the short contact with the standard surface. It can also be described as the force needed to separate a sample from the surface after a short time. The tests were carried out due to the industrial standard FINAT FTM 9—“Loop tack measurement” [20]. A strip of PSA tape is formed in a loop and mounted in the upper grip of the tensile testing machine, and the steel plate is mounted in the lower grip. The loop is contacted briefly with the plate and separated at once, with the force measurement. The measurements were carried out using the same testing machine as described above. The separation rate was 30 cm/min, and the contact area sample-plate was 25 × 25 mm.

#### 2.3.5. Thermal Resistance

Determination of thermal resistance is performed using SAFT (Shear Adhesion Failure Temperature) tests, which were conducted and samples were prepared according to the procedure described for the cohesion tests. A mass of 1 kg was hung at each end of the sample and placed in the programmable oven. The temperature was then slowly increased from 22 to 217 °C at a heating rate of 1 °C/min. The damage temperature was recorded along with the nature of the joint break. The tests were carried out with four samples for each formulation, and the mean value of temperature resistance was calculated.

#### 2.3.6. Shrinkage

The shrinkage test was carried out according to the standard FINAT FTM 14—“Dimensional stability” [20]. A sample constructed from PVC foil rectangle (10 cm × 10 cm) coated with an adhesive film was applied onto an aluminum plate. In the center of the foil, vertical and horizontal incisions (80 mm) were made to form a cross. The sample prepared in this way was conditioned in the thermostatic chamber at 70 °C for a defined time. The width of the slits formed by the cuts was examined with a magnifying glass after 10 and 30 min, respectively; 1, 3, 8, and 24 h; 2, 3, 4, 5, 6, and 7 days.

## 3. Results and Discussion

Table 3 shows the effect of the crystal rock addition on the change in viscosity over time at the highest filler concentration (3 pph) for the tested resin–crosslinker–filler composition. The composition gelled after approximately 10 days—this means that the composition had at least doubled its initial viscosity to a point where it was no longer suitable for coating. The observed changes in viscosity of the tested samples are similar to comparable systems containing silicon resins and other silica fillers [13,21,22]. Comparing the viscosity changes in the resin–filler–crosslinker system and the system without filler, we can observe a faster increase in viscosity over time, but the growth trend is similar. Increase in viscosity over time after addition of quartz filler in comparison to the pot life of neat resin is a very interesting phenomenon. This effect is even more interesting because it concerns very low filler concentrations, unusual for, e.g., microcomposites. The reduction in pot life time can probably be explained by formation of gelation centers due to perfect affinity of silica filler crystallites with the resin. Formation of microcrystallites, as small ordered regions can occur, which become crystallization nuclei, but without possibility of further growth. They probably accelerate the formation of a three-dimensional network and the gelling of the entire composition [23,24,25].

The observed increase in viscosity was uniform throughout the samples, and no sedimentation of filler particles was observed over time; this is most likely related to the maximum amount of the additive (3 pph) and good dispersion of the particles in the silicone resin, while higher filling levels could show a tendency to sedimentation and free agglomeration. Pot life was evaluated for the highest concentration of the filler, due to the influence of filler addition on the viscosity of initial composition—obvious when adding a solid filler to a liquid matrix.

**Figure 2:**
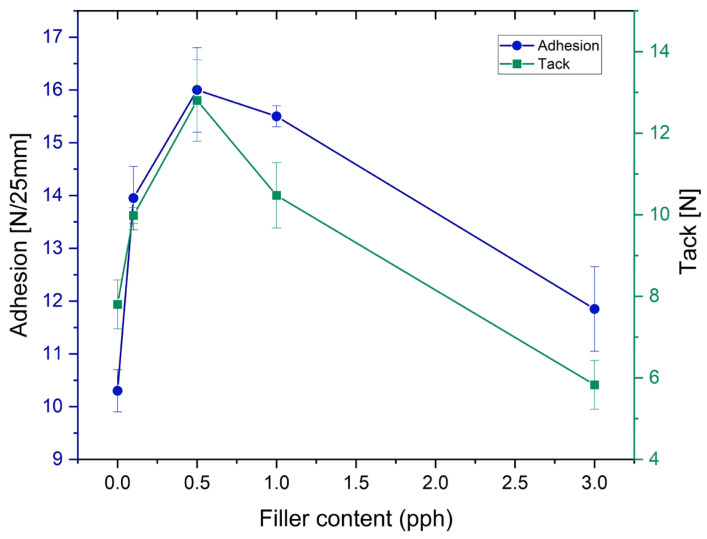
Effect of rock crystal addition on the peel adhesion and tack of silicone pressure-sensitive adhesives.

The effects of the rock crystal addition on properties such as adhesion and tack of self-adhesives tapes made of modified silicone self-adhesive adhesives are shown in Figure 2. For adhesion, an initial increase in the value of both tested properties was observed, until it reached a maximum at approximately 0.5 pph of filling, and then decreased. That effect has been observed in previous studies and also reported in the literature [16,26].

The polymer structure of prepared PSA, tested in conditions far above its Tg, is mainly amorphous. The addition of the fine fraction of mineral filler probably forces the order in the emerging polymer network of the adhesive film and thus influences its viscoelastic properties and decreases tack and adhesion [23,27]. However, peel adhesion values, despite the maximum addition of 3 pph, did not fall below the adhesion level for the pure sample.

The tack values show a similar tendency due to the increase in the concentration of rock crystal. The one difference is that for 3.0 pph of filler content, tack decreases below the level obtained for the neat adhesive sample. This may be caused by the appearance of filler particles on the surface of the adhesive film, which may weaken the tack more than the adhesion itself.

Table 4 describes the results of cohesion tests at room temperature (23 °C), elevated temperature (70 °C), and thermal resistance determined using the SAFT test. Shear strength at 23 °C for all tested samples showed higher values than the tested sample without filler and reached the threshold value required for an industrially produced tape (above 72 h), which clearly indicates an improvement in the cohesive properties of the composition and may be caused by a more rigid structure of the adhesive film. Taking into account the previously described properties of the filler and its influence on the gelation of the prepolymer, as well as the probable ordering of the structure of the adhesive layer during crosslinking [23], similarities can be noticed with the structure of inorganic–organic micro composites [28,29,30,31]. Despite much lower filler concentrations than those used in particle-reinforced composites, it clearly strengthens the internal cohesion of the PSA layer. This is probably due to the good affinity of the silicone resin and the silica filler, which facilitates wetting during mixing, as well as the uniform dispersion of the filler particles in the resin and additionally increased matrix–filler adhesion. Base adhesive used in the studies is Q2-7355, which is composed of polydimethylsiloxane gum and resin. This composition has good compatibility with the silica fillers due to the presence of silanol groups that can interact with polydimethylsiloxane chains. Compatibility of that system can be additionally increased by functionalization of the filler surface, but even pure quartz filler has enough affinity to the polymer matrix [32,33].

Analogous results were obtained for samples tested at 70 °C; they showed similar properties as in the case of tests at room temperature—only the sample with the highest degree of filling (3 pph) showed cohesion below the maximum required test time.

In the SAFT test, which the obtained adhesive tapes were subjected to in order to determine their thermal resistance, most samples achieved very high values, including the two with the lowest degree of filling, which reached the test maximum (225 °C), and the sample containing 1.0 pph, which had values close to it. Only the highest grade sample filled with crystal rock achieved a test value of 152 °C, which is only 20 degrees higher than the reference sample. Decrease in cohesion and SAFT with the increase in filler concentration is very interesting. This may be caused by a disturbance in the adhesion–cohesion balance, e.g., caused by differences in the thermal conductivity of the polymer matrix and the quartz filler crystals, and thus by weakening the adhesion of the filler particles in the matrix. Local aggregation of filler particles at concentrations above 3 pph is also possible, weakening the cohesion of the adhesive layer.

However, the results reveal the increase in general thermal resistance of adhesive with the use of the rock crystal additive with appropriate concentration, along with accepted application properties of obtained PSA samples.

**Figure 3:**
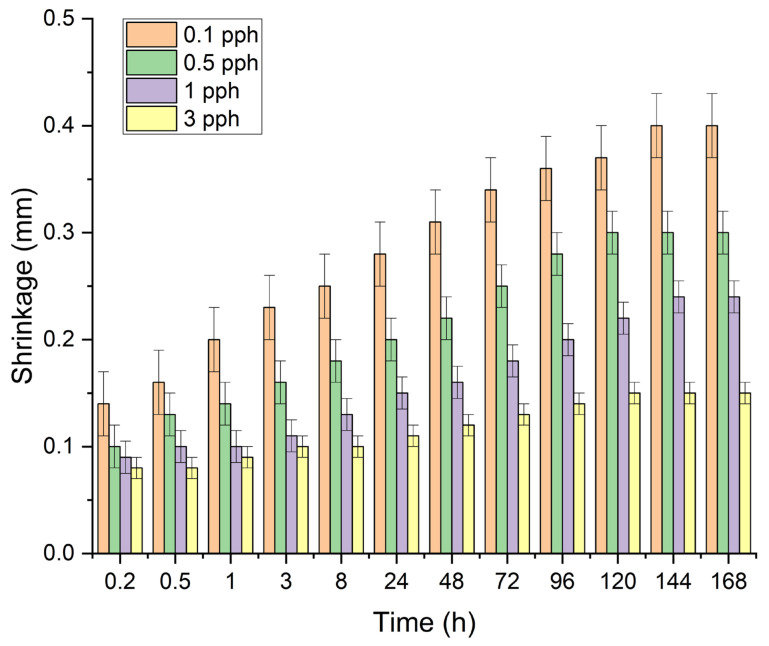
Effect of rock crystal addition on the shrinkage of silicone pressure-sensitive adhesives.

Table 5 and Figure 3 summarize the results of the influence of the amount of rock crystal addition on the shrinkage (dimensional stability) of the modified adhesive film. The tests showed a decrease in its value with an increase in the amount of filler in the polymer matrix of the silicone pressure-sensitive adhesive. This may be due to the previously described better arrangement of the polymer mesh forced by the addition of spatial obstacles in the form of adhesive particles and thus a more compact internal structure of the adhesive film. Each time, contractions stabilized after approximately the 7th day of the study. The lowest values were obtained for a filling with a concentration of 3 pph—0.15%.

## 4. Long-Term Thermal Stability Tests

Pressure-sensitive silicone adhesives can be utilized across a wide temperature range and under conditions characterized by alternating exposure to very low and very high temperatures. Additional modifications with various fillers (e.g., titania, kaolin, vermiculite) can enhance the high-temperature properties of these adhesives, including reducing flammability, as demonstrated in previous studies [15,17,34]. It was also necessary to investigate how low-temperature conditions affect the stability of adhesive joints after a specified period. Following performance tests conducted according to FINAT industrial standards, a decision was made to perform additional tests after conditioning the adhesive joints at low temperature (−20 °C) for silicone PSA samples containing 0.5 pph of the filler. Measurements were carried out in accordance with FINAT standards once the samples reached the appropriate temperature for testing. Two types of samples for conditioning were prepared. First, sample tapes: adhesive coated onto carrier and covered with protective layer. These samples were conditioned in low temperature, and after the planned time, heated to room temperature. Next, they were adhered to standard metal plates in room temperature, according to FINAT standards, and then tested. Second: sample joints tape–metal. Sample tapes were first adhered to metal plates, then cooled to −20 °C, and conditioned for prolonged time. Next, they were heated to standard temperature and tested. For the second type of samples, tack evaluation was not performed.

**Figure 4:**
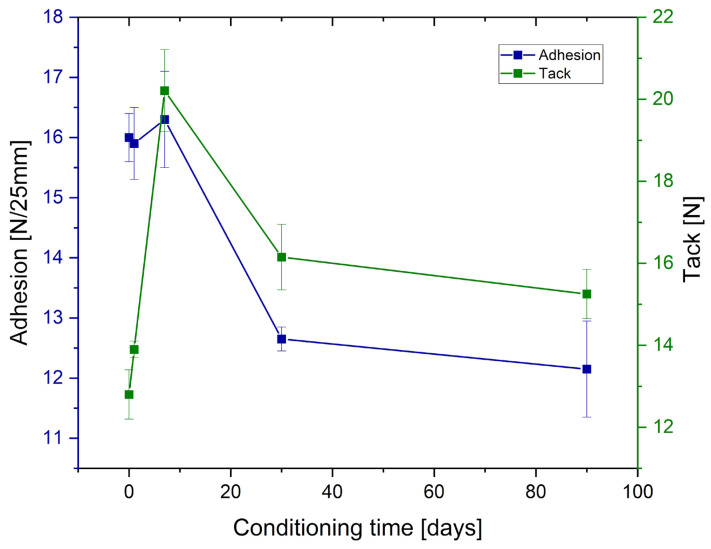
Effect of conditioning in low temperature (−20 °C) of prepared Si-PSAs with 0.5 pph filler content on the peel adhesion and tack of the tape sample.

**Figure 5:**
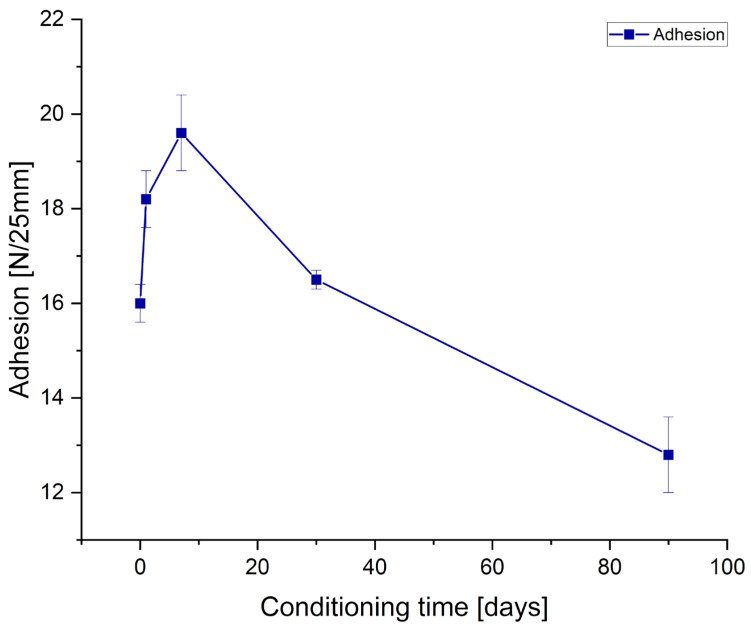
Effect of conditioning in low temperature (−20 °C) of prepared Si-PSAs with 0.5 pph filler content on the peel adhesion of the joint sample.

Adhesion and tack measurements after low-temperature treating of the samples are presented in Figure 4 and Figure 5, for tape and joint samples, respectively.

Cohesion and thermal resistance of low-temperature conditioned samples of Si-PSA with 0.5 pph of the filler are presented in Table 6 and Table 7, for tape sample and joint sample, respectively.

As can be observed from the Figure 4 and Figure 5 and Table 6 and Table 7, prolonged conditioning of the samples significantly reduced the performance parameters of the obtained adhesives. Adhesion experienced a significant decline for both tape samples and tape-to-metal joints. Tack for the tape samples decreased to a lesser extent and, ultimately, was higher than before conditioning; however, this represents an exception among the evaluated parameters. Regarding cohesion measured at room temperature, it can be assumed that freezing had no effect. In contrast, measurements taken at elevated temperature (70 °C) revealed a notable deterioration of the internal cohesion of the adhesive layer following prior freezing. Similarly, SAFT tests indicated a substantial reduction in the thermal resistance of the adhesives (both tapes and joints) after sample conditioning at low temperatures.

## 5. Industrial Application Test

To evaluate the properties of the compositions under industrial conditions, tests were conducted using self-adhesive composites as bonding agents in 3D printing. These compositions were intended to serve as the adhesive material bonding the insulating layer located beneath the printer beds, which isolates the heating cables positioned there and reduces heat loss by directing the thermal energy as desired.

For this purpose, among the received self-adhesive materials, tapes exhibiting the highest thermal resistance were selected (0.5 pph). Subsequently, analogous tapes were prepared for labeling, with the key modification that the compositions were coated onto fluorosilicone-treated release film to enable removal of the carrier after application. Using these tapes, heating cables were arranged beneath the printer bed, and an insulating layer composed of aluminum foil was adhered on top.

In the original configuration, the self-adhesive layer exhibited discoloration, degradation, and loss of adhesive properties after approximately three months, resulting in the detachment of the protective foil and a consequent destabilization of the thermal bed. In contrast, beds protected with adhesive films based on the newly modified compositions maintained stable thermal parameters and showed no signs of deterioration (at the time of writing, 8 months of service). Preliminary inspection of the used self-adhesive material revealed no color changes or degradation.

## 6. Conclusions

The physical modification of industrial silicone resin with the addition of rock crystal was successful. In this way, new self-adhesive materials based on pressure-sensitive adhesive silicones were obtained and characterized. An increase in the value of thermal resistance, an increase in cohesion (at room and elevated temperatures), and a reduction in shrinkage of the obtained new self-adhesive materials, while maintaining an acceptable level of basic properties such as adhesion and tack, were confirmed.

The best results of most important application parameters of PSAs such as tack, peel adhesion, and shear strength (cohesion) were obtained for the samples containing 0.5 pph of rock crystal filler. Also, acceptable (but not the best) shrinkage parameter was achieved for this filler concentration. The cohesion and thermal resistance of manufactured PSA tape samples increased to a high degree. The study reveals that even small amounts of pure quartz filler can significantly improve properties of commercially available PSA resin. Moreover, the studies showed novel interesting effects occurring in rock crystal-reinforced PSA, particularly viscosity changes and cohesion increase, even with a small addition of the inorganic material to the polymer matrix. It is a good point for further studies of adhesion–cohesion balance and filler–matrix interface in silicone pressure-sensitive adhesives.

Additional investigations of the changes in the functional properties of Si-PSA containing 0.5 pph filler, following long-term conditioning of the samples at low temperature, enabled the assessment of the suitability of these adhesives under variable thermal conditions and a wide temperature range. The study revealed that silicone adhesives based on DOWSIL Q2-7355 resin and containing rock crystal as a filler perform significantly better at elevated temperature ranges. However, their prolonged exposure to sub-zero temperatures (−20 °C) substantially reduces their subsequent strength at temperatures above 70 °C.

The exemplary application test of the obtained adhesive under the operating conditions of a 3D-printer-heated bed indicated long-term stability of the adhesive layer. However, quantifying this stability through standard parameter tests such as adhesion, cohesion, and SAFT would require further investigations, which have already been planned.

The best obtained tapes, characterized by increased thermal resistance—above the measurement point—can potentially be used in aviation, heavy and automotive industry, and heating. They will certainly meet the high quality and resistance criteria set for the material used in these industries. The rock crystal used is not an industrially sourced or processed material; it was mechanically ground to obtain the filler. As a natural filler, it has not yet been employed in this form in the industry. This represents a potentially novel application that does not incur costs associated with modifications or specialized filler production, as its acquisition and processing can be limited to mechanical cleaning and grinding.

## Figures and Tables

**Table 1:** Particle size distribution of rock crystal filler.

D_0.5_	D_0.9_	D_3.2_	D_4.3_
[µm]			
8.23 ± 0.38	21.44 ± 0.35	3.692 ± 0.23	10.28 ± 0.32

**Table 2:** Basic properties of selected composition for testing (without filler).

Peel Adhesion [N/25 mm]	Cohesion [h]		SAFT [°C]	Tack [N]	Shrinkage After Stabilization 7 Days [%]
	20 °C	70 °C			
10.8	10.3	3.2	131	7.8	0.27

**Table 3:** Viscosity variation of silicon resin compositions containing 3 pph of crystal rock with storage time, compared to resin without filler.

		Viscosity [Pas]				
Filler Content [pph]	1 Day	2 Days	3 Days	5 Days	7 Days	10 Days
0	16.9	17.3	18.7	22.1	24.9	gel
3	17.1	18.4	20.7	25.5	30.1	gel

**Table 4:** Cohesion and thermal resistance of prepared Si-PSAs with different concentrations of rock crystal filler.

Cohesion and Thermal Resistance					
Filler Content [pph]	0.0	0.1	0.5	1.0	3.0
Cohesion at room temperature [h]	10.3	>72	>72	>72	>72
Cohesion at 70 °C [h]	3.2	>72	>72	>72	64.5
SAFT [°C]	131	>225	>225	218	152

**Table 5:** Shrinkage of silicone pressure-sensitive adhesives with various additions of crystal rock.

Shrinkage (%)												
Cont. of Diatomite (pph)	10 min	30 min	1 h	3 h	8 h	24 h	2 Days	3 Days	4 Days	5 Days	6 Days	7 Days
0.1	0.14	0.16	0.20	0.23	0.25	0.28	0.31	0.34	0.36	0.37	0.40	0.40
0.5	0.10	0.13	0.14	0.16	0.18	0.20	0.22	0.25	0.28	0.30	0.30	0.30
1.0	0.09	0.10	0.10	0.11	0.13	0.15	0.16	0.18	0.2	0.22	0.24	0.24
3.0	0.08	0.08	0.09	0.10	0.10	0.11	0.12	0.13	0.14	0.15	0.15	0.15

**Table 6:** Cohesion and thermal resistance of prepared Si-PSAs with 0.5 pph filler content after conditioning in low temperature (−20 °C) for the tape sample.

Cohesion and Thermal Resistance					
Conditioning Time [Days]	0.0	1	7	30	90
Cohesion at room temperature [h]	>72	>72	>72	>72	>72
Cohesion at 70 °C [h]	>72	21.4	50.2	46.2	10.1
SAFT [°C]	>225	215	159	132	110

**Table 7:** Cohesion and thermal resistance of prepared Si-PSAs with 0.5 pph filler content after conditioning in low temperature (−20 °C) for the joint sample.

Cohesion and Thermal Resistance					
Conditioning Time [Days]	0.0	1	7	30	90
Cohesion at room temperature [h]	>72	>72	>72	>72	>72
Cohesion at 70 °C [h]	>72	65.3	23.0	17.2	9.1
SAFT [°C]	>225	189	181	176	182

## Data Availability

The datasets presented in this article are not readily available because they are also part of an ongoing study. Requests to access the datasets should be directed to A.K.A.
